# Microencapsulation of *Lactobacillus Acidophilus* by Xanthan-Chitosan and Its Stability in Yoghurt

**DOI:** 10.3390/polym9120733

**Published:** 2017-12-20

**Authors:** Guowei Shu, Yunxia He, Li Chen, Yajuan Song, Jiangpeng Meng, He Chen

**Affiliations:** 1 School of Food and Biological Engineering, Shaanxi University of Science and Technology, Xi’an 710021, China; skyheyxkd@gmail.com (Y.H.); skdsongyj@gmail.com (Y.S.); chenhe419@gmail.com (H.C.); 2 College of Food Engineering and Nutritional Science, Shaanxi Normal University, Xi’an 710119, China; 3 Xi’an Baiyue Goat Milk Corp., Ltd., Xi’an 710089, China; byjpmeng@gmail.com

**Keywords:** xanthan-chitosan, encapsulation, *Lactobacillus acidophilus*, RSM, storage stability

## Abstract

Microencapsulations of *Lactobacillus acidophilus* in xanthan-chitosan (XC) and xanthan-chitosan-xanthan (XCX) polyelectrolyte complex (PEC) gels were prepared in this study. The process of encapsulation was optimized with the aid of response surface methodology (RSM). The optimum condition was chitosan of 0.68%, xanthan of 0.76%, xanthan-*L. acidophilus* mixture (XLM)/chitosan of 1:2.56 corresponding to a high viable count (1.31 ± 0.14) × 10^10^ CFU·g^−1^, and encapsulation yield 86 ± 0.99%, respectively. Additionally, the application of a new encapsulation system (XC and XCX) in yoghurt achieved great success in bacterial survival during the storage of 21 d at 4 °C and 25 °C, respectively. Specially, pH and acidity in yogurt were significantly influenced by the new encapsulation system in comparison to free suspension during 21 d storage. Our study provided a potential encapsulation system for probiotic application in dairy product which paving a new way for functional food development.

## 1. Introduction

Probiotics, live microbial supplements, can provide health benefits on the host upon ingestion in a sufficient number [1]. Previous researches proved that probiotics contributed greatly to stronger immunity, lower cholesterol, and blood pressure. Probiotics are involved in improving lactose tolerance, preventing cancer, and Helicobacter pylori infection [2,3,4,5]. The adequate survival of living probiotic should be maintained during shelf-life storage and internal gastro-intestinal tract to benefit human health [6,7]. However, probiotic survival suffers from various food stress factors, such as pH, water activity, oxygen, and competitive microbes [8]. In that case, encapsulation of probiotic cells attracts extensive attention, where bacteria cells are entrapped in varieties of encapsulating agents to avoid undesirable loss during storage and passage through the gastro-intestinal tract [9,10,11].

Among various bio-polymeric encapsulation systems, the xanthan-chitosan mixture has been identified as high potential hydrogel system for targeted delivery and controlled release of encapsulated products for oral administration [12,13]. Chitosan is the natural cationic polysaccharide generated by alkaline deacetylation of chitin, which carries positive charges at pH values below 6.5. Xanthan gum is an anionic polyelectrolyte consisting of a cellulosic backbone with side chains of two mannoses and one glucuronic acid on every second glucose residue. For this reason, the amino groups of chitosan and carboxyl groups of xanthan gum can form a three-dimensional network through the ionic interactions with the properties of low toxicity, high resistance to enzyme and pH-sensitive swelling [14,15]. The xanthan–chitosan polyelectrolyte complex (PEC) gels are mostly applied to enzyme microcapsules currently [12,16]. However, the study about encapsulation of *L. acidophilus* in xanthan–chitosan (XC) and xanthan-chitosan-xanthan (XCX) PEC gels is scarce. Therefore, encapsulation of *L. acidophilus* in XCX and XC PEC gels by the extrusion method was investigated in our study.

In our previous research, the effects of chitosan concentration and pH, xanthan concentration, cell suspension-xanthan ratios, XCL/chitosan ratios on *L. acidophilus* microcapsules have been studied by single-factor tests. The Plackett-Burman experiment showed that chitosan concentration, xanthan concentration, and XLM/chitosan were three main factors for the viable counts of *L. acidophilus* microcapsules [17]. In this study, the preparation condition of *L. acidophilus* microcapsules (XC) was optimized by response surface methodology (RSM). A three-level, three-factor Box–Behnken design was employed to study the effects of chitosan concentration, xanthan concentration, and XLM/chitosan on the viable count and entrapped yield of *L. acidophilus* microcapsules. Furthermore, the effect of the optimized encapsulation system (XC and XCX) on the survival of *L. acidophilus* in yoghurt was studied at 4 °C and 25 °C, respectively.

## 2. Materials and Methods

### 2.1. Preparation of Xanthan and Chitosan Solutions

Xanthan (Zhongxuan biological chemistry Co., Ltd., Shandong, China) with a molecular weight of 1.02 million was dissolved in deionized water to the concentration of 0.72%, 0.75%, 0.78% (*w/v*). Then the solution was sterilized with moist heat at 110 °C for 10 min, cooled and centrifuged at 7000 rpm for 10 min. Chitosan (Xingcheng Biological Co., Ltd., Jiangsu, China) with a molecular weight of 0.37 million were dissolved into stirring HCl (1 mol·L^−1^) solution to the concentration of 0.64%, 0.67%, 0.70% (*w/v*). And NaOH (1 mol·L^−1^) and deionized water were added to adjust the pH of chitosan solution to 5.5.

### 2.2. Microorganism

*L. acidophilus*, isolated from commercial dairy product, was preserved in the lab of School of Food and Biological Engineering, Shaanxi University of Science and Technology. *L. acidophilus* were inoculated in MRS medium and incubated at 37 °C for 24 h. The cells were harvested by centrifugation at 10,000 rpm for 15 min and re-suspended in 0.9% sterilized saline water. 

### 2.3. Encapsulation Procedure and Encapsulation Yield

The extrusion technique was applied for production of *L. acidophilu* microcapsulation. *L. acidophilus* culture was dispersed in xanthan solution (1:10) thoroughly. The mixture was dripped into chitosan solution placed on magnetic stirrer through a manually operated syringe with 0.7-mm cannula. The chitosan solution was stirred constantly to allow crosslinking of the capsules. Then wet capsules were filtered and washed by sterilized saline water for 3 times, the XC beads loaded with *L. acidophilus* was obtained [18]. Then resultant microcapsules were suspended to 0.1% (*w/v*) xanthan solution, after being stirred for 30 min on the magnetic stirrer, the double microcapsules (XCX) were prepared for further analysis.

KH_2_PO_4_ (6.8 g) was dissolved in 250 mL of deionized water, NaOH (0.2 mol·L^−1^) was used to adjust the pH of solution to 7.5. Trypsin (10 g) was dissolved in 400 mL deionized water and mixed with the above mentioned KH_2_PO_4_ solution. The final volume was adjusted to 1 L with deionized water, the simulated intestinal juice was prepared. One-gram microcapsules were dispersed in 10 mL of simulated intestinal juice, after being oscillated at 37 °C for 40 min under 210 rpm. The encapsulation yield was calculated according to Equation (1):

(1) $${\mathrm{EY}\;=\;(N}_{1}\cdot M)/\left(N_{0}\cdot V_{0}\right)\;\times 100\%$$

where N_1_ (CFU·mL^−1^) was viable counts of microcapsules after subjected to simulated intestinal juice. M (g) was the weight of the wet microcapsule. N_0_ (CFU·mL^−1^) was the initial viable counts in the cell suspension. V_0_ (mL) was the volume of original bacteria liquid using for microcapsule.

### 2.4. Box-Behnken Design (BBD) and Statistical Analysis

Based on the results of single factor test and Plackett–Burman experiment, the process of *L. acidophilus* microcapsules (XC) was further optimized by a three-factor, three-level Box-Behnken model in this study. The three effective factors, chitosan concentration (A), xanthan concentration (B), XLM/chitosan were independent variables. Three levels in the coded (−1, 0, 1) and actual factors levels were shown in Table 1.

### 2.5. Storage Stability of Free Cells, Optimized L. acidophilus Encapsulation (XC and XCX) in Yoghurt

3% starter culture (*Streptococcus thermophilus* and *Lactobacillus bulgaricus*) were inoculated into pure milk, after being cultivated at 25 °C for 24 h, the yoghurt was prepared. XC and XCX microcapsules were incorporated into yoghurt (pH of 4.5) made by our laboratory in the ratio of 1:9 (*w/v*) respectively. A control group was done by adding 1 mL free *L. acidophilus* suspension to 9 mL of yoghurt. Then the mixture was stored at 4 °C and 25 °C respectively. Viable counts, acidity and pH were determined in the stipulated time (0, 1, 3, 7, 14 and 21 d) to know the changes of these indexes [18]. Each group was conducted three parallel experiments. The survival of free and encapsulated cells was measured by the plate count method as described in [17]. MRS agar containing 0.06% bile salt was used to determine viable counts of *L. acidophilus* in yoghurt. The acidity (°T) was determined by the method of neutralization titration according to the GB5413.34-2010 (National food safety standard: Determination of acidity in milk and milk products). The pH of triplicate sample was recorded using a pH-meter (pHS-3C Shanghai Precision Scientific Instrument Co., Ltd., Shanghai, China). 

## 3. Results and Discussion

### 3.1. Optimization of Encapsulation Conditions by RSM

#### 3.1.1. Effect of Chitosan Concentration, Xanthan Concentration, and XLM/Chitosan on Viable Counts

A Box-Behnken design was employed to investigate the optimal combination of three independent variables, chitosan concentration (A), xanthan concentration (B), and XLM/chitosan (C). A total of 15 runs of BBD experiment design and the results of response value Y_1_ (viable counts) and Y_2_ (encapsulation yield) according to RSM are listed in Table 2.

Based on the experimental result of Table 2, Design-Expert (V8.0.6) software was applied in the experiment for regression analysis and model fitting. Each variable and response value were describing by following multiple regression equations:

(2) $$Y_{1}\;=\;13.28\;+\;0.41A\;+\;0.61B-0.58C\;+\;0.25\mathrm{AB}\;+\;0.40\mathrm{AC}-\mathrm{BC}\;-\;0.90A^{2}-1.95B^{2}-1.33C^{2}$$

where Y_1_ is the viable counts of *L. acidophilus* microcapsules (CFU·g^−1^), A, B, and C represent chitosan concentration (%, *w/v*), xanthan concentration (%, *w/v*), and XLM/chitosan, respectively.

The effectiveness of the regression equation and the significance level of the model were assessed by an Analysis of Variance (ANOVA) test. The *p* value could test the significance of each coefficient and reveal the interaction pattern of independent variables [19]. According to the result of ANOVA in Table 3, the *p* value for the model was 0.0003, indicating the high significance of the model, and the *p* value of 0.1261 (*p* > 0.05) for lack of fit proved the adequacy of the regression model. What’s more, the *p* values of factors A, B, and C were less than 0.05, showing significant effect on the viable counts. And the order of variables affecting viable counts was as follows: xanthan concentration (B) > XLM/chitosan (C) > chitosan concentration (A). The *p* values of factors A^2^, B^2^, and C^2^ were less than 0.01, showing that the correlation between response value and variables were not a simple linear relation. The goodness-of-fit of model was expressed by the coefficient of determination (*R*^2^ = 96.51%), suggesting that only 3.49% of the total variations was not explained by the response model. The value of adjustment coefficient (*R*_adj_^2^ = 92.02%) close to *R*^2^ indicated a satisfactory relation between the measured value and the predicted value of viable counts. 

**Figure 1:**
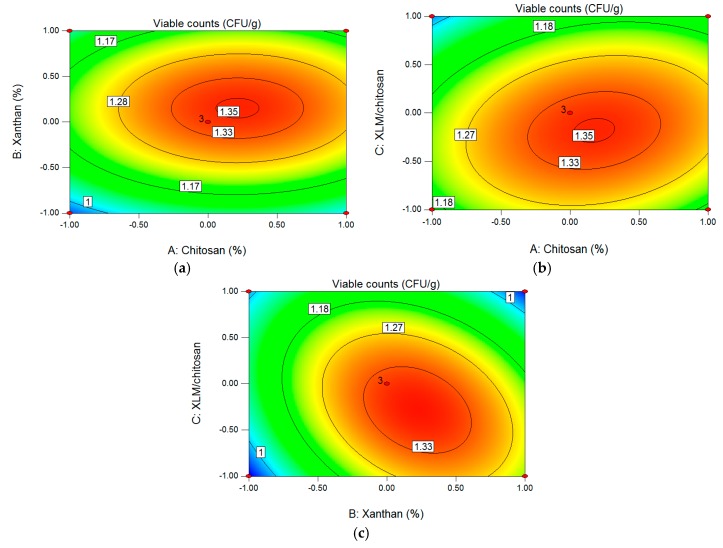
Contour plot and response surface plot showing interaction effects of A (chitosan concentration) and B (xanthan concentration) (**a**), A (chitosan concentration) and C (XLM/chitosan) (**b**), B (xanthan concentration) and C (XLM/chitosan) (**c**) on the viable counts (Y_1_).

The two-dimensional contour plot can describe whether the mutual interactions between the independent variables (chitosan concentration, xanthan concentration, XLM/chitosan) are significant or not. [20]. Elliptical contour plots exhibited significant interaction between the independent variables, while round contour plots implied that the interactions between independent variables are non-significant [21]. For chitosan concentration (A), xanthan concentration (B) (Figure 1a), the contour plot was close to being a circle, showing weak mutual interaction between parameters A and B. The same trend was observed between parameters A and C (Figure 1b), the mutual interaction between chitosan concentration (A) and XLM/chitosan (C) was weak, indicating that the mutual interaction between chitosan concentration and XLM/chitosan was not significant. For xanthan concentration (B) and XLM/chitosan (C) (Figure 1c), the contour plot was oval obviously, showing strong mutual interaction between parameters B and C, which fit well with the results of interaction terms in variance analysis. 

#### 3.1.2. Effect of Chitosan Concentration, Xanthan Concentration, and XLM/Chitosan on Encapsulation Yield 

Design-Expert (V8.0.6) software was applied to analyze the data in Table 2. Response value Y_2_ (encapsulation yield) was described by Equation (3):

(3) $$Y_{2}\;=\;88.06\;+\;2.60A\;+\;3.84B-0.34C\;+\;4.15\mathrm{AB}-0.40\mathrm{AC}\;-\;6.23\mathrm{BC}-6.52A^{2}-13.34B^{2}-6.89C^{2}$$

where Y_2_ is the encapsulation yield of *L. acidophilus* microcapsules (%), A, B, and C represents chitosan concentration (%, *w/v*), xanthan concentration (%, *w/v*), XLM/chitosan, respectively.

An Analysis of Variance (ANOVA) was employed to evaluate the statistical significance of the developed models, the results were shown in Table 4.

From the results (Table 4), the low *p* value of 0.0013 of the model with an *F* value of 21.86 indicated that regression model was significant. The *p* value of lack of fit was 0.0862 higher than 0.05, which was non-significant. The independent variables with the greatest effect on encapsulation yield was B (xanthan concentration), followed by variable A (chitosan concentration) and the quadratic term coefficients of B^2^, C^2^ and A^2^. And the coefficient of determination (*R*^2^) was 96.56%, indicating that the response model could explain 96.56% of the total variability. And the value of adjustment coefficient (*R*_adj_^2^) was 92.15%, indicating that there was high fitting degree between measured and predicted values of encapsulation yield, which proved the feasibility of the experiment method.

**Figure 2:**
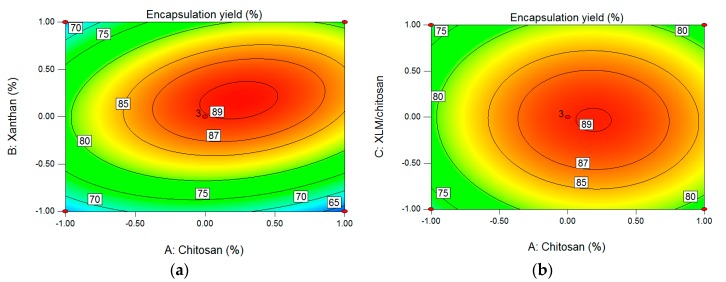

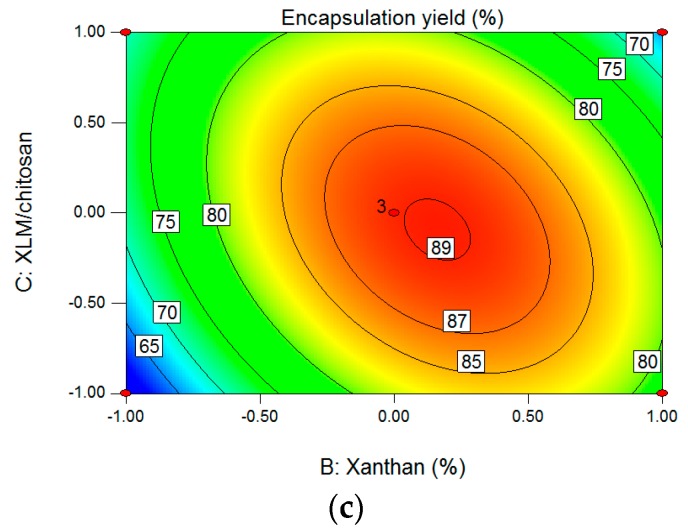
Contour plot and response surface showing interaction effects of A (chitosan concentration) and B (xanthan concentration) (**a**), A (chitosan concentration) and C (XLM/chitosan) (**b**), B (xanthan concentration) and C (XLM/chitosan) (**c**) on encapsulation yield (Y_2_).

For A (chitosan concentration) and B (xanthan concentration) (Figure 2a), the oval contour plot implied that there was strong mutual interaction between parameters A and B. The circle contour plot in Figure 2b showed that the mutual interaction between chitosan concentration and XLM/chitosan was non-significant. The results in Figure 2c were similar to the findings of Figure 2a the contour plot was oval obviously, implying strong mutual interaction between parameters B (xanthan concentration) and C (XLM/chitosan), which was consistent with the results of interaction terms in Table 4.

#### 3.1.3. Verification of the Model

Considering the results of the regression model, response surface plots, and contour plots, the optimum encapsulation condition was a chitosan concentration of 0.68%, a xanthan concentration of 0.76%, and an XLM/chitosan of 1:2.56. Under optimal conditions, the maximum predicted values of response Y_1_ (viable counts) and Y_2_ (encapsulation yield) were 1.35 × 10^10^ CFU·g^−1^ and 88%. In order to measure and verify the optimized result, three confirmatory experiments were carried out under the optimum conditions (chitosan concentration 0.68%, xanthan concentration 0.76%, XLM/chitosan 1:2.56). The experimental viable counts of *L. acidophilus* microcapsules was 1.31 ± 0.14 × 10^10^ CFU·g^−1^, and the corresponding encapsulation yield was 86 ± 0.99% (mean ± SD). There were no significant differences between predicted values and measured values, which demonstrated the reliability of experiment method.

### 3.2. Storage Stability of Encapsulated L. acidophilus (XC and XCX) in Yogurt

**Figure 3:**
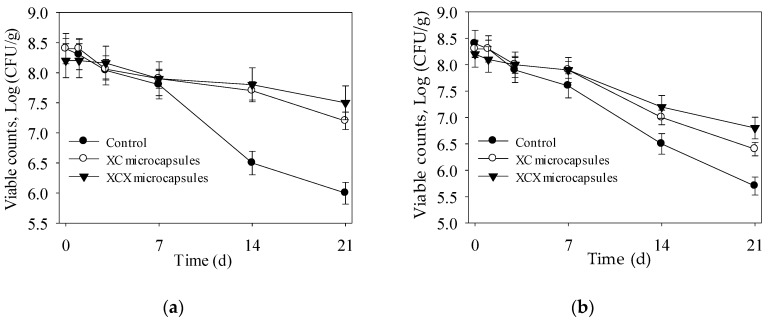
Survival of encapsulated and free *L. acidophilus* incorporated into yogurt during storage at 4 °C (**a**) and 25 °C (**b**). ● Free *L. acidophilus*; ○ XC microcapsules; ▼ XCX microcapsules. The error bars represent standard deviation of means (*n* = 3).

Probiotic microcapsules were usually incorporated into yoghurt, resulting in lower post-acidification and higher survival during its shelf life [22,23]. Bacterial loss both happened in encapsulated and non-encapsulated *L. acidophilus* in yoghurt through a 3 weeks storage period at 4 °C and 25 °C (Figure 3). After storage at 4 °C for 21 days, the XC and XCX microcapsules showed 1.2 log (from 8.4 to 7.2 log) and 0.7 log (from 8.2 to 7.5 log) CFU·mL^−1^ loss in cell number, respectively (Figure 3a), while there was 2.4 log decrease (from 8.4 to 6 log CFU·mL^−1^) in the number of free cells. These results showed that XCX microencapsulation achieved significant success in bacterial improvement when compared to non-encapsulated cells. Similarly, Soma [24] reported that encapsulation of *L. acidophilus* ATCC 43121 in XCX hydrogel capsules in stirred yogurt resulted in higher viability during refrigerated storage conditions. And the final numbers of XC and XCX microcapsules were 7.2 and 7.5 log CFU·mL^−1^, which are still beyond the threshold level of therapeutic requirement (10^6^–10^7^ CFU·g^−1^) in yoghurt.

As shown in Figure 3b, there was a sharp decline in the number of free cells in yoghurt at 25 °C. After 3 weeks storage, the XC and XCX microcapsules showed 0.8 log and 1.3 log CFU·mL^−1^ preservation in cell number over the free *L. acidophilus*. These results were consistent with results studied by Heidebach [25], which indicated that encapsulated strains in hydrocolloid gels can increase their viability by 0.5–3 log cycles CFU in yoghurt during its shelf life. The XC and XCX microcapsules kept the viable counts above the requirement level for 14 days at 25 °C in yoghurt.

**Figure 4:**
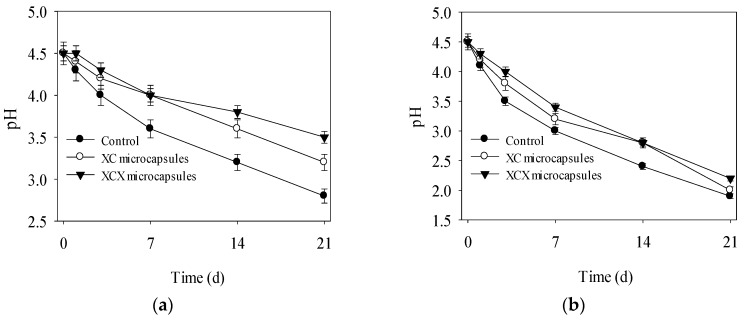

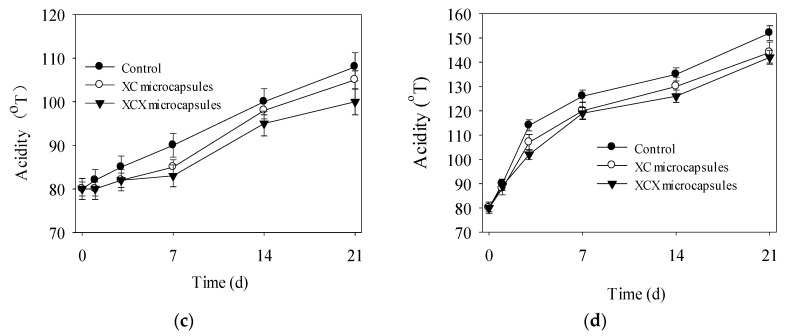
The pH of encapsulated and free *L. acidophilus* incorporated into yogurt during storage at 4 °C (**a**) and 25 °C (**b**); Acidity of encapsulated and free *L. acidophilus* incorporated into yogurt during storage at 4 °C (**c**) and 25 °C (**d**).● Free *L. acidophilus*; ○ XC microcapsules; ▼ XCX microcapsules. The error bars represent standard deviation of means (*n* = 3).

Figure 4 depicts the changes of pH and acidity of yoghurt during the 21 d storage. Declining pH and rising pH were observed in yoghurts at both 25 °C and 4 °C. The yoghurt with free cells showed a reduction of 2.7 in pH, where the final pH was down to 2.8, whereas higher final pH was observed in yoghurt containing XC and XCX microcapsules (Figure 4a). Over 21 d storage at 4 °C, the acidity of yoghurt containing XC and XCX microcapsules increased by 25 °T and 20 °T, while the yoghurt with free cells showed an increment of 28 °T (Figure 4c). The lower post-acidification in yoghurt with encapsulated cells might be explained by that the XC and XCX encapsulations lead to lower metabolic activity of microorganisms [18,26]. Similar results were reported by Kailasapathy [27], who proved that yoghurt containing *L. acidophilus* encapsulated in calcium alginate matrix exhibited less post-acidification than yoghurts with free cells. More acidity increment and pH reduction were displayed at 25 °C (Figure 4b,d). These might be attributed to vigorous metabolism of *L. acidophilus* at 25 °C and the acidogenic fermentation of yogurt. 

Considering the pH, acidity and viable counts of final products, the optimal storage time of yoghurt with free, XC and XCX microcapsules was 7 d, 14 d and 21 d at 4 °C and 3 d, 7 d and 14 d at 25 °C, respectively. The number of probiotic bacteria was maintained above 10^7^ CFU·mL^−1^ in yoghurt during its shelf life. These results demonstrated that XC and XCX microcapsule both enhanced the stability of *L. acidophilus* in yoghurt during the storage period at 4 °C and 25 °C, respectively.

## 4. Conclusions

In the present study, *L. acidophilus* was immobilized with XC and XCX hydrogels by extrusion. A three-factor, three-level Box-Behnken model was employed to optimize the process of *L. acidophilus* microencapsulation in XC hydrogels. The optimal parameters were chitosan of 0.68%, xanthan of 0.76%, and XLM/chitosan of 1:2.56. Under these conditions, the mean experimental value of encapsulation yield and the viable count of *L. acidophilus* microcapsules were 86 ± 0.99% and (1.31 ± 0.14) × 10^10^ CFU·g^−1^, respectively, which were close to the predicted value of 88% and 1.35 × 10^10^ CFU·g^−1^. In addition, XC and XCX microcapsules showed less post-acidification and higher viability in yoghurt. The new encapsulation system (XC and XCX) provided an extension of shelf-life of yoghurt at both 4 °C and 25 °C. In conclusion, microcapsule of either XC or XCX, could serve as a good encapsulation system for yoghurt contained *L. acidophilus*, and becoming a potential technology in the development of probiotic functional foods.

## Figures and Tables

**Table 1:** The factors level coding table of Box-Behnken Design of *Lactobacillus acidophilus* microcapsules.

Variable	Level		
	−1	0	1
A Chitosan (%)	0.64	0.67	0.70
B Xanthan (%)	0.72	0.75	0.78
C XLM/chitosan	1:2.4	1:2.6	1:2.8

**Table 2:** The experimental design and results of Box-Behnken of *L. acidophilus* microcapsules.

RUN	A	B	C	Y_1_ (10^10^ CFU·g^−1^)	Y_2_ (%)
1	−1	1	0	1.03	67.5
2	1	−1	0	1.05	60.6
3	0	1	1	0.90	61.8
4	0	−1	1	0.99	70.3
5	1	0	−1	1.13	76.9
6	0	0	0	1.37	90.1
7	0	0	0	1.35	89.6
8	0	1	−1	1.21	77.8
9	1	1	0	1.19	80.3
10	1	0	1	1.09	78.3
11	0	0	0	1.31	86.9
12	−1	−1	0	0.90	64.4
13	−1	0	1	1.0	73.2
14	−1	0	−1	1.20	70.2
15	0	−1	−1	0.90	61.4

**Table 3:** The ANOVA of *L. acidophilus* microcapsules Box-Behnken with viable counts (Y_1_) as response value.

Source	SS	DF	MS	*F* Value	*p* Value	Sig
Model	41.32	9	4.59	21.49	0.0003	***
A	1.36	1	1.36	6.37	0.0396	*
B	3.00	1	3.00	14.05	0.0072	**
C	2.64	1	2.64	12.38	0.0097	**
AB	2.5 × 10^−3^	1	2.5 × 10^−3^	0.012	0.9169	-
AC	0.64	1	0.64	3.00	0.1271	-
BC	4.00	1	4.00	18.72	0.0035	*
A^2^	3.43	1	3.43	16.05	0.0051	**
B^2^	16.05	1	16.05	75.13	<0.0001	***
C^2^	7.42	1	7.42	34.73	0.0006	***
Error	1.50	7	0.21	-	-	-
Lack of fit	1.09	3	0.36	3.55	0.1261	-
Pure error	0.41	4	0.10	-	-	-
Total	42.82	16	-	-	-	-

DF: Degree of freedom; SS: sum of squares; MS: mean square; * *p* < 0.05; ** *p* < 0.01; *** *p* < 0.001; *R*^2^ = 96.51%; *R*_adj_^2^ = 92.02%

**Table 4:** The ANOVA of *L. acidophilus* microcapsules Box-Behnken with encapsulation yield (Y_2_) as response value.

Source	SS	DF	MS	*F* Value	*p* Value	Sig
Model	1637.36	9	181.97	21.86	0.0013	**
A	54.08	1	54.08	6.50	0.0382	*
B	117.81	1	117.81	14.15	0.0071	**
C	0.91	1	0.91	0.11	0.7504	-
AB	68.89	1	68.89	8.28	0.0238	*
AC	0.64	1	0.64	0.077	0.7896	-
BC	155.00	1	155.0	18.62	0.0035	**
A^2^	178.85	1	178.85	21.48	0.0024	**
B^2^	749.57	1	749.57	90.04	<0.0001	***
C^2^	200.03	1	200.03	24.03	0.0017	**
Error	58.27	7	8.32	-	-	-
Lack of fit	45.26	3	15.09	4.64	0.0862	-
Pure error	13.01	4	3.25	-	-	-
Total	1696.04	16	-	-	-	-

DF: Degree of freedom; SS: sum of squares; MS: mean square; * *p* < 0.05; ** *p* < 0.01; *** *p* < 0.001; *R*^2^ = 96.56%; *R*_adj_^2^ = 92.15%.
